# Changes in the Physicochemical Properties of Blood and Skin Cell Membranes as a Result of Psoriasis Vulgaris and Psoriatic Arthritis Development

**DOI:** 10.3390/ijms21239129

**Published:** 2020-11-30

**Authors:** Izabela Dobrzyńska, Barbara Szachowicz-Petelska, Adam Wroński, Iwona Jarocka-Karpowicz, Elżbieta Skrzydlewska

**Affiliations:** 1 Faculty of Chemistry, University in Białystok, Ciołkowskiego 1K, 15-245 Białystok, Poland; basia@uwb.edu.pl; 2 Dermatological Specialized Center “DERMAL” NZOZ in Białystok, Nowy Swiat 17/5, 15-453 Białystok, Poland; adam.wronski@dermal.pl; 3 Department of Analytical Chemistry, Medical University of Białystok, Mickiewicza 2, 15-230 Białystok, Poland; iwona-jarocka-karpowicz@umb.edu.pl (I.J.-K.); elzbieta.skrzydlewska@umb.edu.pl (E.S.)

**Keywords:** psoriasis, blood cells, skin cells, electrical properties, membrane composition

## Abstract

Psoriasis is accompanied by disturbed redox homeostasis, with systemic and local oxidative stress promoting the modification of basic components of cellular membranes. Therefore, the aim of the study was to investigate the effect of development of psoriasis vulgaris and psoriatic arthritis on the composition and physicochemical properties of skin cell membranes (keratinocytes and fibroblasts) and blood cells (lymphocytes, granulocytes and erythrocytes). Both forms of psoriasis are characterized by decreased levels and changes in the localization of membrane phospholipids, and an increased level of sialic acid as well as the lipid peroxidation product (malondialdehyde), which resulted in an increase in the zeta potential of skin cells and blood cells, with granulocytes and lymphocytes affected more than erythrocytes. Using theoretical equations and the dependence of the cell membrane surface charge density as a function of pH, it was shown that patients with psoriatic arthritis have a greater increase in the concentration of negatively charged groups on the membrane surface and reduced the value of the association constant with H^+^ compared to patients with psoriasis vulgaris. Therefore, it can be suggested that the physicochemical parameters of membranes, skin and blood cells, especially lymphocytes, can be used to assess the severity of the disease.

## 1. Introduction

Psoriasis is an autoimmune and chronic inflammatory disease with symptoms mainly involving pathophysiological changes in the skin and joints. In addition to metabolic changes, the symptoms of psoriasis cause considerable psychosocial disability and significantly affect the quality of life of patients [1]. The essence of the disease process is the excessive number of cell divisions in the basal layer of the epidermis and the accelerated, abnormal maturation cycle of keratinocytes. In addition, the pathological interactions between immune cells (especially lymphocytes) and skin cells (especially keratinocytes) are characteristic of psoriasis. Some patients with psoriasis experience a more severe clinical course leading to the development of psoriatic arthritis [2,3].

Psoriasis is believed to be caused by a combination of environmental and genetic factors leading to excessive activation of immune cells and metabolic changes related to the development of a pro-inflammatory phenotype [4,5]. The consequence is redox imbalance and the occurrence of both local and systemic oxidative stress that activates leukocytes and increases inflammation and increased activation of lymphocytes by dendritic cells, which is considered a key stage in the development of autoimmune diseases [6,7,8]. Thus, the development of psoriasis is mediated by dendritic cells as well as lymphocytes and leukocytes. In contrast, inflammatory cytokines such as interleukin-23 (IL-23) and interleukin-6 (IL-6) produced by dendritic cells and macrophages facilitate the differentiation of TH17 cells, which secrete IL-17 and other mediators that stimulate epidermal cell proliferation and contribute to aberrant keratinocyte differentiation [9]. Moreover, pro-inflammatory signaling pathways such as MAP kinase activated protein kinase (MAPK/AP-1), nuclear factor kappa-light-chain-enhancer of activated B cells (NFkB) and Janus kinase and signal transducer and activator of transcription (JAK-STAT) are activated in cells involved in the development of psoriatic lesions [10,11,12]. Previous studies have shown elevated levels of NFkB and pro-inflammatory cytokines in both skin and blood cells (granulocytes and lymphocytes) [6,7,13]. It is therefore believed that clinical symptoms are directly caused by immune cell activity, with changes in biochemical parameters, including those associated with redox imbalance, and consequently oxidative stress, affecting both skin and blood cells, including granulocytes, lymphocytes and erythrocytes of patients with psoriasis [5,6,14,15]. Oxidative conditions favor oxidative modification of cellular components, mainly proteins and lipids, both in skin and blood cells of patients with psoriasis [6,7,14,15,16,17,18,19,20]. In addition, a positive correlation between the markers of oxidative stress and the area of psoriasis and the disease severity index was shown [21].

Recent reports indicate that the consequence of oxidative stress is increased lipid metabolism, especially the metabolism of membrane phospholipids, containing polyunsaturated fatty acids (PUFAs), in cells (both skin and blood) of patients with psoriasis, which is dependent on both reactive oxygen species (ROS) and enzymes [6,7,14,15,18,19]. Consequently, increased levels of oxidative fragmentation and cyclization products of PUFAs are observed [6]. Among other compounds, α,β-unsaturated reactive aldehydes can act as secondary messengers of radical reactions and regulatory signaling molecules that, by modifying cellular metabolism, can even lead to cell death by apoptosis [22]. In particular, among the immunologically competent inflammatory cells of patients with psoriasis, granulocytes generate lipid peroxidation products, thereby modulating their proinflammatory activity [6]. The activation of lymphocytes is also accompanied by the generation of large amounts of ROS as well as the overproduction of cytokines that activate other immune cells, leading to chronic inflammation [23].

In inflammation, due to the increased activity of phospholipid-metabolizing enzymes (phospholipase, cyclooxygenase, and lipoxygenase), the enzyme-dependent metabolism of membrane phospholipids is also intensified [24]. The endocannabinoids generated indirectly through the activation of cannabinoid receptors modulate redox processes and inflammatory processes, since the activation of cannabinoid receptor type 1 (CB1) receptors promotes the increased generation of ROS and tumor necrosis factor-α, and the activation of cannabinoid receptor type 2 (CB2) receptors inhibits the generation of ROS and the abovementioned proinflammatory cytokine [25,26]. Moreover, the increased production of eicosanoids, under the influence of cyclooxygenases and lipoxygenases, depending on the type of compounds generated, can model the inflammatory process in a different way [6,27,28]. The abovementioned reactions intensify the modification of the phospholipid composition of the cell membranes, resulting in changes in the physicochemical properties, including the electrical properties, of these membranes [29,30,31]. The value of the electrical charge of the cell membrane can therefore be a source of information about the equilibrium, or lack thereof, between the membrane and its surroundings as well as about physiological and pathophysiological conditions such as psoriasis. Thus, monitoring the zeta potential and the electric charge of the cell membrane as a function of pH as well as the parameters characterizing the membrane, such as the total concentrations of negatively (C_TA_) and positively (C_TB_) charged groups and their association constants with H^+^ (K_AH_) and OH^−^ (K_BOH_) ions in the context of modifying the levels of phospholipids, sialic acid, and lipid peroxidation products (e.g., malondialdehyde, MDA) of the cell membranes of blood and skin cells could help to assess the severity of lesions in psoriasis patients.

Therefore, the aims of this study were to determine the effects of psoriasis vulgaris and psoriatic arthritis on lipid peroxidation, the content of membrane components (e.g., phospholipids and sialic acid), and, consequently, the electrical properties of the cell membranes of skin cells (keratinocytes and fibroblasts) and blood cells (erythrocytes, lymphocytes and granulocytes).

## 2. Results

### 2.1. Membrane Components

The development of the disease affects the levels of phospholipids. Table 1 shows a significant reduction in the amounts of phospholipid (phosphatidylcholine (PC), phosphatidylethanolamine (PE), phosphatidylserine (PS), and phosphatidylinositol (PI)) in the skin cells (keratinocytes and fibroblasts) from the patients with psoriasis vulgaris. Similarly, Table 2 presents a reduction in the same phospholipid levels in the blood cells (erythrocytes, lymphocytes, and granulocytes) from the patients with psoriasis vulgaris or psoriatic arthritis, compared with the healthy subjects. Additionally, a stronger decrease in these phospholipid levels was observed in the cells from the patients with psoriasis vulgaris.

**Figure 1:**
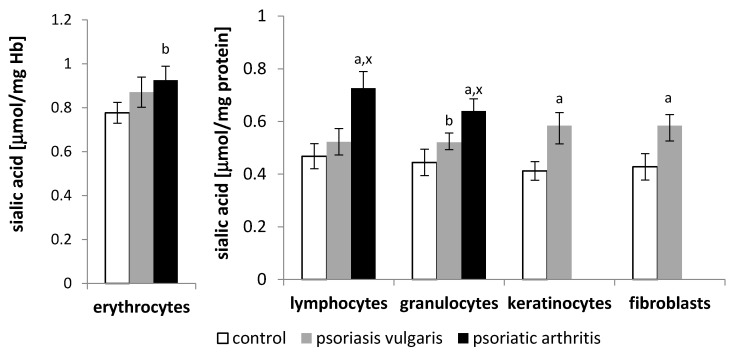
The sialic acid content in blood cells (erythrocytes, lymphocytes and granulocytes) as well as skin cells (keratinocytes and fibroblasts) of patients with psoriasis vulgaris (*n* = 16) and psoriatic arthritis (*n* = 8) as well as healthy subjects (*n* = 8). Data points represent the mean ± SD; a—statistically significant differences vs. healthy subjects, *p* < 0.0001; b—statistically significant differences vs. healthy subjects, *p* < 0.05; x—statistically significant differences vs. patients with psoriasis vulgaris, *p* < 0.01.

The development of psoriasis also caused changes in the level of sialic acid (Figure 1), which is one of the basic components of glycoproteins and glycolipids contained in the cell membrane. The keratinocytes, fibroblasts, lymphocytes, and granulocytes, but not erythrocytes, from the patients with psoriasis vulgaris had higher sialic acid levels than those from the healthy subjects. In contrast, the patients with psoriatic arthritis had increased levels of sialic acid in all examined blood cell types. A much larger increase in the amount of sialic acid was observed in the lymphocytes from the patients with psoriatic arthritis than those with psoriasis vulgaris.

### 2.2. Lipid Peroxidation

**Figure 2:**
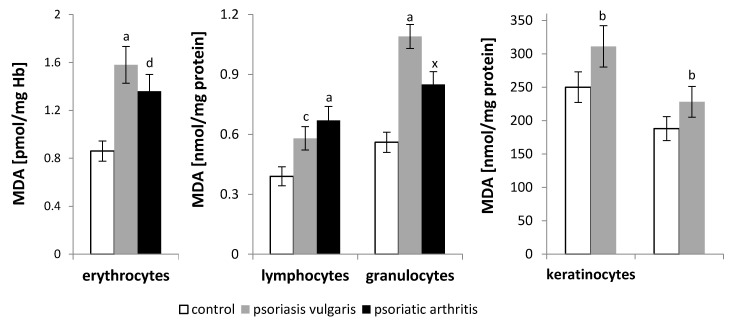
The level of lipid peroxidation product (malondialdehyde (MDA)) in blood cells (erythrocytes, lymphocytes and granulocytes) as well as skin cells (keratinocytes and fibroblasts) of patients with psoriasis vulgaris (*n* = 16) and psoriatic arthritis (*n* = 8) as well as healthy subjects (*n* = 8). Data points represent the mean ± SD; a—statistically significant differences vs. healthy subjects, *p* < 0.0001; b—statistically significant differences vs. healthy subjects, *p* < 0.001; c—statistically significant differences vs. healthy subjects, *p* < 0.01; d—statistically significant differences vs. healthy subjects, *p* < 0.05; x—statistically significant differences vs. patients with psoriasis vulgaris, *p* < 0.05

An increase in the level of malondialdehyde (MDA), which belongs to the group of low-molecular-weight aldehydes formed during oxidative PUFA fragmentation, was observed in both the psoriasis vulgaris and psoriatic arthritis patients (Figure 2), and it corresponded to changes in the membrane phospholipid contents in the individual cell types. Larger increases in the MDA level were observed in the erythrocytes and granulocytes of the patients with psoriasis vulgaris and in the lymphocytes of the patients with psoriatic arthritis, compared to those of the healthy controls.

### 2.3. Physicochemical Properties

**Figure 3:**
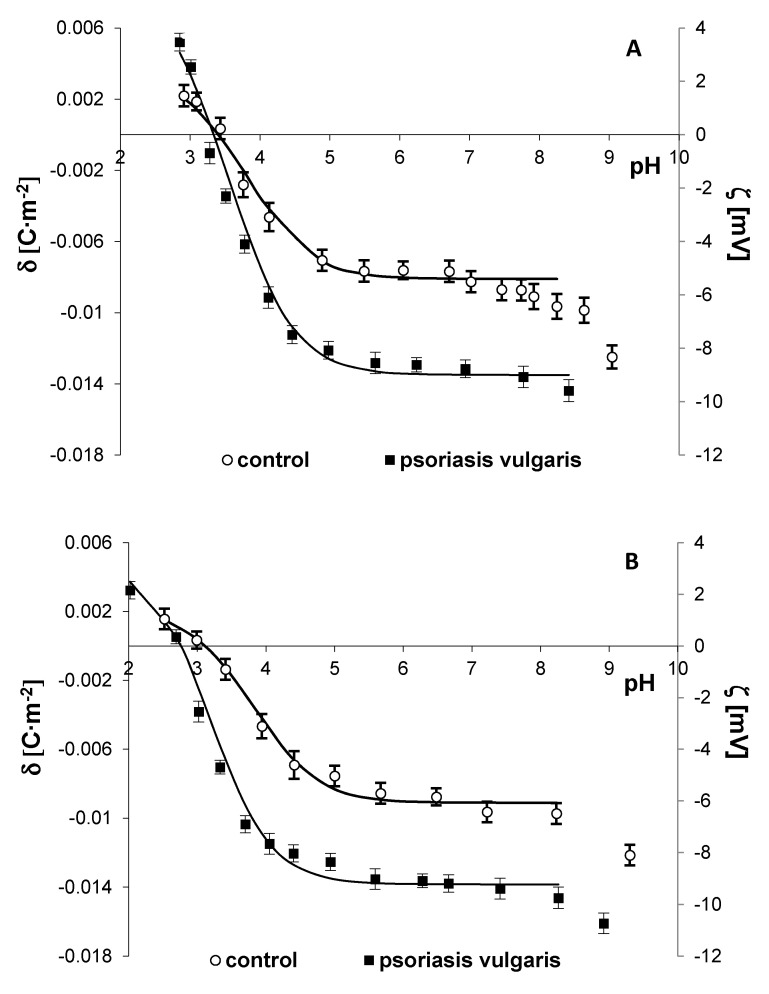
The surface charge density (left axis) and corresponding zeta potential (right axis) of keratinocyte (**A**) and fibroblasts (**B**) cell membrane of patients with psoriasis vulgaris (*n* = 16) as well as healthy subjects (*n* = 8). Data points represent the mean ± SD.

**Figure 4:**
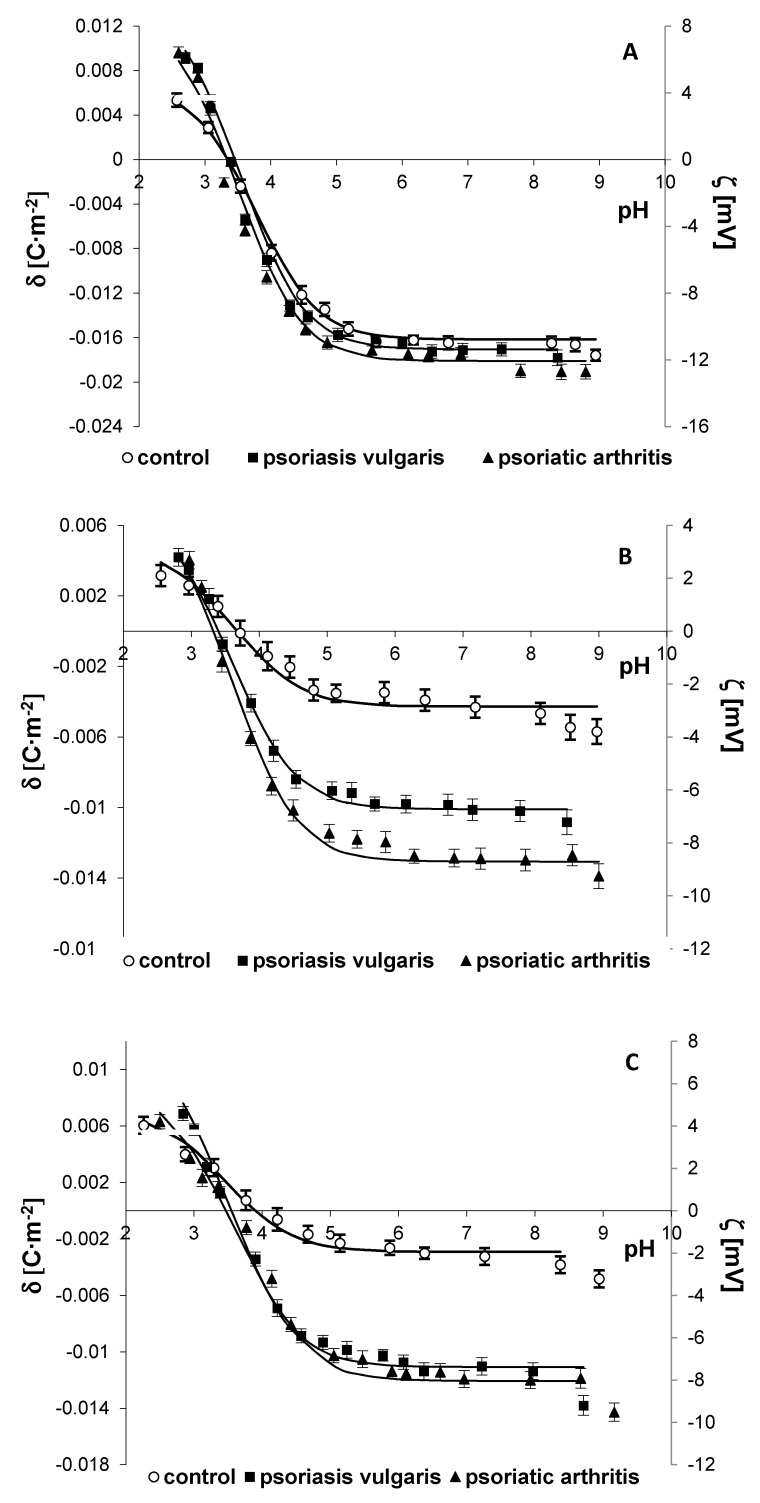
The surface charge density (left axis) and corresponding zeta potential (right axis) of erythrocyte (**A**), lymphocyte (**B**) and granulocyte (**C**) cell membranes of patients with psoriasis vulgaris (*n* = 16) and psoriatic arthritis (*n* = 8) as well as healthy subjects (*n* = 8). Data points represent the mean ± SD.

Figure 3 and Figure 4 present the surface charge density and the zeta potential, respectively, of the cell membranes of keratinocytes, fibroblasts, erythrocytes, lymphocytes, and granulocytes as a function of pH. The experimental values are marked with points, and the theoretical values are denoted as a continuous line.

Only the cell membranes of keratinocytes, fibroblasts, lymphocytes, and granulocytes from the patients with psoriasis vulgaris were characterized by an increased negative charge at high pH values, compared to the cells from healthy subjects. In contrast, an increase in the negative membrane charge was observed in all examined cells from the patients with psoriatic arthritis. In the case of lymphocytes, more changes were observed in the cells from the patients with psoriatic arthritis than from those with psoriatic vulgaris. In addition, the isoelectric points of the cell membranes of keratinocytes, lymphocytes, and granulocytes from the patients with psoriasis vulgaris or psoriatic arthritis were shifted towards lower pH values, compared to the healthy controls. Most likely, this effect was caused by an increased number of acidic groups with a smaller association constant located in the cell membranes of these cells.

Mathematical calculations based on a model describing the adsorption of electrolyte ions on the surface of the cell membrane enabled quantitative assessment of the parameters characterizing the membrane (C_TA,_ C_TB,_ K_AH_, and K_BOH_). The determined constants were substituted in Equation (2), providing theoretical curves. An agreement between the theoretical and experimental surface charge densities was observed (Figure 3 and Figure 4). Increases in C_TA_, C_TB_, and K_BOH_ and a decrease in K_AH_ for the cell membranes of keratinocytes, lymphocytes, and granulocytes were observed in both the psoriasis vulgaris patients and the psoriatic arthritis patients (Table 3), compared to the controls. Changes in the composition of the functional groups of the cell membrane may result from the appearance or disappearance of new groups during ongoing modifications during the course of the disease.

## 3. Discussion

The cell membrane is an essential element for the proper functioning of individual cells in addition to tissues and the whole body. The cell membrane integrity ensures proper cell metabolism as well as communication and intercellular transport [32,33]. The cell membrane is also responsible for the processes of cell proliferation and differentiation, the disorder of which is especially observed in the keratinocytes of psoriasis patients [34]. It is believed that this may be the result of disturbed membrane phospholipid homeostasis, including biosynthesis and metabolism, mainly in epidermal cells such as keratinocytes, but also in primary dermal cells such as fibroblasts [34,35,36], which was confirmed by the results of this work. However, psoriasis is a systemic disease that manifests not only changes at the level of skin cells, but it is also associated with dysfunction of many organs, which leads to an increased risk of cardiovascular diseases [37]. Therefore, the metabolic assessment of blood cells is important for assessing the state of human health, including responses from all cells/tissues of the body.

The development of psoriasis is accompanied by inflammation and oxidative stress, which are related to, inter alia, the interaction of two important transcription factors: Nrf2, which is responsible for regulating the biosynthesis of cytoprotective proteins, including antioxidants, and nuclear factor κB, which is responsible for regulating the biosynthesis of proinflammatory cytokines that play an important role in the pathogenesis of this disease [14,38]. In patients with psoriasis, it is known that neutrophils, which are also involved in the production of proinflammatory chemokines, are mainly responsible for the production of ROS [39]. Especially in psoriatic arthritis, the number of circulating neutrophils is significantly increased, which leads to higher levels of ROS [40]. The simultaneous reduction in the cell antioxidant capacity causes a redox homeostasis disorder, leading to a redox imbalance [6,41].

The prooxidative conditions observed in psoriasis favor modifications of the basic components of the cell membrane, including phospholipids, proteins, glycolipids, and glycoproteins [42,43]. According to the literature, the development of psoriasis is accompanied by disorders of phospholipid metabolism resulting from both the overproduction of ROS and the increased activity of enzymes responsible for the metabolism of cell membrane phospholipids [6]. Phospholipase A2 (PLA2) activity is increased, but it is much higher in patients with psoriasis vulgaris compared to patients with psoriatic arthritis [6,35]. In contrast, an increased PLA2 activity leads to an increased release of PUFAs, including arachidonic acid, which is further metabolized mainly by the participation of cyclooxygenases and lipoxygenases [27,28]. Consequently, it has been demonstrated that the development of psoriasis is accompanied by changes in the biosynthesis of various lipid mediators, including endocannabinoids and eicosanoids [6,7]. Elevated levels of the two major endocannabinoids (anandamide and 2-arachidonoyl glycerol) have been observed in patients with both forms of psoriasis, despite the increased activity of enzymes degrading these endocannabinoids (e.g., fatty acid amide hydrolase and monoacylglycerol lipase) [7]. It is believed that endocannabinoids, which are agonists of specific G protein-coupled receptors, have pleiotropic effects, depending on the type of activated receptor, and thus model redox and inflammatory processes [7]. In this way, they control lipid metabolism as well as the proliferation, differentiation, and apoptosis of skin cells [44].

The intensification of metabolism of both free and phospholipid PUFAs depends on ROS as well as enzymes, consequently leading to reduced phospholipid levels in both skin cells and blood cells, especially in lymphocytes and granulocytes, which was observed in this work. It has been hypothesized that a decrease in the PC level, which was observed in this study, may be caused by an increase in lecithin-cholesterol acyltransferase activity, which leads to the formation of lysophosphatidylcholine (LPC) and ceramides [45]. In addition, PC deacylation by the PC–sphingomyelin (SM) transacylase occurs, resulting in a decreased PC level and an increased SM level [35]. In contrast, an increased level of SM in keratinocytes from patients with psoriasis is accompanied by a decreased level of ceramides, which are responsible for the basic functions of the skin, including the permeability barrier in the intercellular space and water retention in the stratum corneum [46,47]. It has been reported that the levels of phospholipid metabolism products (e.g., lysoglycerophospholipids), such as lysophosphatidylcholine (LPC) and lysophosphatidic acid (LPA), as well as the metabolism of glycerophospholipids, including PA, PC, and PI, are significantly altered in the plasma of patients with psoriasis [48]. In addition, it has been observed that the LPC levels are significantly increased in the plasma of psoriasis patients, compared to the PC levels [48]. In contrast, LPC exerts a metabolic effect by participating in signaling pathways in cells such as T lymphocytes, monocytes, and neutrophils [49]. LPC and LPA are metabolically the most significant lysoglycerophospholipids and are considered inflammatory lipids involved in the development of immune diseases, including psoriasis [50]. However, the reduction in PI levels in the epidermis of patients with psoriasis may be associated with the increased phosphorylation of PI by phosphatidylinositol 3-kinase [51], and overexpression of phosphatidylinositol 3-kinase may play an important role in the pathogenesis of psoriasis by mediating immunopathogenesis, hyperplasia epidermis, and/or angiogenesis [52].

The results of this study indicate that patients with severe psoriasis vulgaris or psoriatic arthritis have an increased ROS-dependent phospholipid metabolism. As a consequence, there is an increase in the level of lipid peroxidation products both in skin cells (keratinocytes and fibroblasts) and blood cells (lymphocytes, granulocytes and erythrocytes) of patients with psoriasis vulgaris and in blood cells (lymphocytes, granulocytes and erythrocytes) which can only be obtained from patients with psoriatic arthritis, the changes of which correspond to the reduced level of phospholipids in the examined cells. By introducing polar peroxide, ketone, aldehyde, or hydroxyl groups into the lipid bilayer, the resulting lipid peroxidation changes the physical properties, including the electrical properties, of the cell membranes [53,54]. In particular, there is a reduced hydrophobicity of the lipid interior of the cell membrane as well as changes in membrane asymmetry, causing disorders in cell signaling that lead to apoptosis [32,55]. This process is accompanied by the appearance of PS on the outer surface of the membrane, which is responsible for the recognition of apoptotic cells by macrophages, followed by their removal by phagocytosis [56]. The above changes as well as the change in cholesterol also reduce the fluidity of the blood cell membrane, especially in the hydrophobic core [36,57,58], where the severity of these changes is directly proportional to the severity of the disease process [36]. Therefore, a decrease in the membrane fluidity corresponds to exacerbation of the changes caused by the development of both forms of psoriasis.

Regardless of qualitative/quantitative changes in phospholipid structure, the development of severe psoriasis vulgaris is accompanied by an increase in the total level of sialic acid (free, as well as glycolipids and glycoproteins) present in the cell membranes of skin cells (keratinocytes and fibroblasts), as well as lymphocytes and granulocytes of patients with psoriasis vulgaris. The same direction of changes in the level of total sialic acid, but with greater intensity, is observed in lymphocytes and granulocytes of patients with severe psoriatic arthritis. According to the literature, patients with psoriasis have an increased expression of the gene responsible for coding the type II membrane protein, a type of sialyltransferase, which catalyzes the transfer of sialic acid to glycolipids to produce the gangliosides GD3 and GT3 [59,60,61]. In contrast, the sialic acid present in gangliosides plays a key role in cell proliferation and migration [62,63]. In addition, glycoproteins with sialic acid residues as well as total sialic acid are considered stable markers of inflammation and free radical scavengers, so they reflect the severity of disease, including psoriasis. This is especially true for the sialic acid content of lymphocytes, which are an important part of the immune system.

Sialic acid, due to its strategic location on the outer surface of the cell membrane, can interact with sialic acid-binding immunoglobulin-type lectins (siglecs), which are key to immune homeostasis [64,65]. Siglecs can bind to the sialic acid or glycoproteins present in another cell or to sialic acid exposed on the same cell, thus affecting cell function, including that of immune cells [66]. These receptor–sialic acid interactions can participate in cell adhesion, cell signaling, and other cell functions [59,67,68]. It has been shown that disorders involving the sialic acid–siglec interaction favor the development of inflammatory diseases [69,70]. Thus, a significantly higher level of sialic acid in the lymphocytes of psoriasis patients indicates an increase in inflammation, especially in those with psoriatic arthritis.

The altered lipid structure and increased level of sialic acid included in the glycolipids and glycoproteins of the cell membranes of skin cells and blood cells in psoriasis vulgaris and in the blood cells of patients with psoriatic arthritis lead to a change in the number of functional groups on the membrane, consequently causing changes in its electrical properties. This mechanism was confirmed by our results. In individuals suffering from psoriasis, an increased negative charge was observed on the surface of the tested cells, compared to the cells of healthy people, with larger changes observed in the psoriatic arthritis patients. An increase in the negative charge on the cell membrane surface can be caused by an increase in the sialic acid content, which was also confirmed by our results, as well as the translocation of anionic phospholipids, especially PS, to the outer part of the cell membrane [71]. These changes were accompanied by increases in the C_TA_, C_TB_, and K_BOH_ values as well as a decrease in the K_AH_ value on the membrane surface. Changes in the constant values suggest that negative groups appearing on the surface show stronger acidic properties. Consequently, a shift in the isoelectric point of the cell membrane towards lower pH values was observed. As in other inflammatory diseases, these modifications affect the membrane permeability and various cell functions, including transport and receptor functions as well as signal transduction [7,47,68,72,73]. As a result, these modifications compromise the cell integrity and increase the possibility of contact with the environment.

## 4. Materials and Methods

### 4.1. Samples for Analysis

Blood cells from psoriasis vulgaris patients and psoriatic arthritis patients as well as skin cells from psoriasis vulgaris patients were used in this study. In addition, blood cells and skin cells were obtained from healthy individuals as controls.

Thirty-two patients (14 females and 18 males, mean age: 41 years old) suffering from psoriasis vulgaris and 16 patients (8 females and 8 males, mean age: 48 years old) suffering from psoriatic arthritis for at least 6 months were qualified for this study. The patients with psoriasis vulgaris were included in this study if their lesions affected at least 10% of their total body surface. The severity of psoriasis was assessed using the psoriasis index and severity index (range: 15–25; median 19). Patients with psoriatic arthritis were diagnosed based on the CLASsification criteria for Psoriatic Arthritis (CASPAR) questionnaire. The control group consisted of 16 healthy subjects (7 females and 9 males, mean age: 43 years old). The major exclusion criteria for all groups were as follows: receiving topical or oral medications during the 4 weeks before the study, comorbidities, and smoking or alcohol abuse. All participants gave their informed consent for inclusion in this study. The research was carried out in accordance with the Helsinki Declaration, and the protocol was approved by the Local Bioethics Commission at the Medical University of Białystok, Poland (No. R-I-002/289/28.09.2017).

Blood samples were collected into ethylenediaminetetraacetic acid (EDTA) tubes, and then two-stage centrifugation was carried out. In the first stage, the samples were centrifuged at 3000× *g* and 4 °C to obtain the plasma and the buffy coat. Erythrocytes, lymphocytes, and granulocytes were isolated from the buffy coat by gradient centrifugation using Gradisol G/L (1:5 ratio). The samples were layered on Gradisol and subjected to centrifugation at 300× *g* for 25 min at room temperature. The individual cell fractions were collected. The antioxidant butylhydroxytoluene was added to the erythrocyte, lymphocyte, and granulocyte samples before storing them to prevent oxidation [6,74].

Skin fragments were taken for histopathological examination (hematoxylin–eosin staining). The remaining samples were washed with phosphate-buffered saline (PBS) containing 50 U/mL penicillin and 50 μg/mL streptomycin and incubated overnight at 4 °C with dispase (1 mg/mL) to separate the epidermis from the dermis. Next, the epidermis was digested with trypsin/EDTA at 37 °C for 20 min, and the separated keratinocytes were washed and resuspended in PBS containing a proteasome inhibitor mix [35].

The dermis was sliced and placed in culture plates containing fibroblast culture medium consisting of Dulbecco’s modified Eagle medium, fetal bovine serum (10%), and penicillin (50 U/mL)/streptomycin (50 μg/mL). The samples were incubated in a humidified atmosphere of 5% CO_2_ at 37 °C until the fibroblasts emigrating from the slices reached full confluence. The fibroblasts were collected from the plates by scraping on ice and suspended in Tris-HCl buffer (50 mM, pH 7.5, 4 °C) containing 0.1% sodium dodecyl sulfate and protease inhibitor cocktail. All samples were lysed by sonification on ice. The total protein content in the cell lysates was measured using the Bradford assay [35,75].

### 4.2. Isolation and Analysis of Phospholipids

Total phospholipids were extracted according to the Folch method [76]. A 1:2 chloroform/methanol mixture was added to the cell membranes of skin cells (keratinocytes and fibroblasts) and blood cells (erythrocytes, lymphocytes, and granulocytes). The mixture was vortexed well. An additional volume of chloroform was added, followed by Milli-Q water. In all steps, the mixtures were strongly vortexed. Finally, the samples were centrifuged at 4000× *g* for 20 min at room temperature to obtain a two-phase system: an aqueous upper phase and an organic lower phase. The total phospholipid extract was recovered from the organic phase into a new tube and dried under a flow of nitrogen. After drying, the total phospholipid extract was resuspended in 200 μL of 1:2 chloroform/methanol. Separation of the phospholipids was achieved by high-performance liquid chromatography (HPLC) (Hitachi, Tokyo, Japan). The phospholipids were separated by group analysis using normal-phase HPLC with a silica gel column. A 130:5:1.5 (*v/v/v*) acetonitrile/methanol/85% phosphoric acid mixture was used as the eluent with isocratic elution at a flow rate of 1 mL/s and a detection wavelength of 214 nm [31].

### 4.3. Determination of the Sialic acid Level

The modified Svennerholm’s resorcinol method was used to determine the total sialic acid content in the membranes of blood and skin cells [77]. The color intensity was measured at 630 nm using a diode array spectrophotometer (Hewlett Packard, Waldbronn, Germany). The sialic acid concentration was determined from the standard curve of the *N*-acetylneuraminic acid solution.

### 4.4. Estimation of Lipid Peroxidation

Lipid peroxidation was estimated by measuring the MDA level. The aldehyde level was measured by gas chromatography tandem mass spectrometry, as the *O*-(2,3,4,5,6-pentafluoro-benzyl)-oxime-trimethylsilane (*O*-PFB-oxime-TMS) derivative, using a modified method of Luo et al. [78]. Benzaldehyde-D_6_, as an internal standard, was added to the cell lysates, and the aldehyde was derivatized by the addition of *O*-PFB-hydroxyamine hydrochloride (0.05 M in piperazine-*N*,*N*′-bis(2-ethanesulfonic acid) buffer, 200 µL) and incubated at room temperature for 60 min. After incubation, the samples were deproteinized by the addition of 1 mL of methanol, and the *O*-PFB-oxime aldehyde derivative was extracted by the addition of 2 mL of hexane. The top hexane layer was transferred into a borosilicate tube and evaporated under a stream of argon gas, followed by the addition of *N,O*-bis(TMS)-trifluoroacetamide in 1% trimethylchlorosilane. A 1-µL aliquot was injected onto the column. The derivatized aldehyde was analyzed using a 7890A GC-7000 quadrupole tandem mass spectrometer (Agilent Technologies, Santa Clara, CA, USA) equipped with a HP-5ms capillary column (0.25 mm internal diameter, 0.25 µm film thickness, 30 m length) and detected in selected ion monitoring mode. The ions used were as follows: *m*/*z* 204.0 and 178.0 for MDA-PFB-TMS and *m*/*z* 307.0 for the internal standard derivative [29,79].

### 4.5. Electrochemical Methods

The electrophoretic mobility and zeta potential of the cell membranes of blood and skin cells were determined using Zetasizer Nano ZS apparatus (Malvern Instruments, Malvern, UK), as described previously [30]. Then, the surface charge density was determined from the electrophoretic mobility using the following formula:

(1) $$\delta =\frac{\eta \cdot u}{d}$$

where u is the electrophoresis mobility, η is the viscosity of the solution, and d is the diffuse layer thickness. The diffuse layer thickness was determined from the formula $d=\sqrt{\frac{\varepsilon \cdot \varepsilon_{0}\cdot R\cdot T}{2\cdot F^{2}\cdot I}}$ where ε·ε_0_ is the permeability of the electric medium, R is the gas constant, T is the temperature, F is the Faraday constant (96,487 (C∙mol^−1^)), and I is the ionic strength of 0.9% NaCl.

The relationships between the surface charge density of the cell membranes of blood and skin cells as well as the pH of the electrolyte solution were described using the mathematical equations provided by Dobrzyńska et al. [80]. This model assumes the existence of four membrane surface equilibria with H^+^, OH^−^, Na^+^, and Cl^−^ ions, with the following final equation describing the surface charge density of the membrane (δ):

(2) $$\frac{\delta }{F}=\frac{C_{\mathrm{TB}}\cdot a_{H^{+}}}{a_{H^{+}}\left(1+K_{\mathrm{BCl}}\cdot a_{{\mathrm{Cl}}^{-}}\right)+{\;K}_{\mathrm{BOH}}\cdot K_{w}}-\;\frac{C_{\mathrm{TA}}}{K_{\mathrm{AH}}\cdot a_{H^{+}}+{\;K}_{\mathrm{ANa}}\cdot a_{{\mathrm{Na}}^{+}}+1\;}$$

where C_TA_ is the total surface concentration of the acidic groups, C_TB_ is the total surface concentration of the basic groups, and K_AH_, K_ANa_, K_BOH_, and K_BCl−_ are the association constants.

Introducing the results of the measurements of the electrical charge dependence of the cell membrane as a function of pH to the theoretical equations that were derived to describe the charge dependence on the solution composition, the total concentrations of C_TA_ and C_TB_ and their association constants with K_AH_ and K_BOH_ were determined. The mathematical calculations based on the model describing the adsorption of electrolyte ions on the cell membrane surface enabled quantitative assessment of the parameters characterizing the membrane (C_TA_, C_TB_, K_AH_, and K_BOH_). The determined constants were substituted into Equation (2), resulting in theoretical curves. The theoretical values for the zeta potential were determined from the following formula [29]:

(3) $$\xi =\frac{3\cdot \delta \cdot d}{2\cdot \varepsilon \cdot \varepsilon_{0}\cdot f\left(\kappa a\right)}$$

where a is the particle radius and κ^−1^ is the Debye length.

### 4.6. Statistical Analysis

The data are expressed as average ± SD. The statistical analysis for the two analyzed groups (it concerns keratinocytes and fibroblasts from healthy humans and psoriasis patients) was performed using Mann–Whitney test. For the three groups (blood cells), data were analyzed by Kruskal–Wallis test with post hoc Dunn’s multiple comparisons tests for multiple comparisons to identify significant differences between groups, including red blood cell, lymphocyte, and granulocyte studies. *p* Values < 0.05 were considered significant (See Supplementary Materials). Statistical analyses were performed using GraphPad Prism for Windows version 7.0.0 (GraphPad software, San Diego, CA, USA).

## 5. Conclusions

Summarizing, the results of this study suggest that the development of severe psoriasis vulgaris is associated with modifications of the composition of cell membranes of skin cells (keratinocytes and fibroblasts), but also of blood cells (granulocytes, lymphocytes and erythrocytes). On the other hand, the development of severe psoriatic arthritis is accompanied by even more severe changes in the composition of blood cells membranes than in psoriasis vulgaris. As a consequence, this leads to the changes in the physicochemical properties of these membranes, especially in patients with psoriatic arthritis.

The greatest changes were observed in lymphocytes, which confirm the special role of these cells in the development of psoriasis. Therefore, it may be suggested that the assessment of physicochemical properties, in particular of the membranes of skin cells and lymphocytes, may help diagnose psoriasis vulgaris, while changes in lymphocytes may be particularly useful in the diagnosis of psoriatic arthritis. It should be emphasized that the changes observed in blood cells that are relatively available for examination may be particularly useful in diagnostics. However, it should not be forgotten that similar changes can also be observed in other inflammatory diseases (including autoimmune diseases such as systemic lupus erythematosus and rheumatoid arthritis), in which metabolic changes may elicit a response similar to that seen in psoriasis. Therefore, evaluation of the lymphocyte membrane response in these diseases is essential before proposing a new diagnostic tool. At the same time, taking into account the possibilities of clinical and pathomorphological assessment of psoriasis, it should be emphasized that the assessment of the physicochemical parameters of cell membranes may only be an element of auxiliary diagnostics in ambiguous situations, especially in psoriatic arthritis. It should also be taken into account that this type of assessment will require both non-standard equipment and properly prepared staff.

## Figures and Tables

**Table 1:** The level of phospholipid fractions (phosphatidylinositol (PI), phosphatidylserine (PS), phosphatidylethanolamine (PE), phosphatidylcholine (PC)) in keratinocytes and fibroblasts of patients with psoriasis vulgaris (*n* = 16) as well as healthy subjects (*n* = 8).

Analysed Parameters	Healthy Subjects (Control)	Psoriasis Vulgaris	
**Keratinocytes (µg/mg protein)**			
PI	1.11 ± 0.12	0.66 ± 0.14 ^a^	a; *p* < 0.0001
PS	1.64 ± 0.21	0.95 ± 0.18 ^a^	a; *p* < 0.0001
PE	3.35 ± 0.35	2.38 ± 0.33 ^a^	a; *p* < 0.0001
PC	3.99 ± 0.41	3.05 ± 0.38 ^a^	a; *p* < 0.0001
**Fibroblasts (µg/mg protein)**			
PI	1.73 ± 0.12	1.45 ± 0.15 ^a^	a; *p* < 0.0001
PS	2.76 ± 0.25	2.04 ± 0.28 ^a^	a; *p* < 0.0001
PE	5.83 ± 0.52	4.81 ± 0.49 ^a^	a; *p* = 0.0009
PC	7.03 ± 0.68	5.72 ± 0.60 ^a^	a; *p* = 0.0012

Data points represent the mean ± SD; a—statistically significant differences vs. control group.

**Table 2:** The levels of phospholipid fractions (PI, PS, PE, PC) in erythrocytes, lymphocytes and granulocytes of patients with psoriasis vulgaris (*n* = 16) and psoriatic arthritis (*n* = 8) as well as healthy subjects (*n* = 8).

Analysed Parameters	Healthy Subjects (Control)	Psoriasis Vulgaris		Psoriatic Arthritis	
**Erythrocytes (µg/mg Hb)**					
PI	1.70 ± 0.16	1.45 ± 0.17 ^a^	a; *p* = 0.0318	1.57 ± 0.19	
PS	2.38 ± 0.17	2.13 ± 0.23 ^a^	a; *p* = 0.0145	2.24 ± 0.21	
PE	2.95 ± 0.24	2.46 ± 0.25 ^a^	a; *p* = 0.0005	2.68 ± 0.27	
PC	7.37 ± 0.45	6.49 ± 0.42 ^a^	a; *p* = 0.0017	6.82 ± 0.45	
**Lymphocytes (µg/mg protein)**					
PI	1.47 ± 0.11	1.20 ± 0.12 ^a^	a; *p* = 0.0006	1.24 ± 0.12	
PS	2.27 ± 0.25	1.71 ± 0.16 ^a^	a; *p* < 0.0001	1.94 ± 0.15	
PE	7.50 ± 0.71	4.90 ± 0.51 ^a^	a; *p* < 0.0001	5.10 ± 0.44 ^a^	a; *p* = 0.012
PC	9.26 ± 0.96	6.90 ± 0.73 ^a^	a; *p* < 0.0001	7.27 ± 0.81 ^a^	a; *p* = 0.0182
**Granulocytes (µg/mg protein)**					
PI	2.13 ± 0.28	1.05 ± 0.24 ^a^	a; *p* < 0.0001	1.11 ± 0.16 ^a^	a; *p* = 0.0085
PS	3.11 ± 0.35	1.34 ± 0.14 ^a^	a; *p* < 0.0001	1.68 ± 0.18 ^x^	x; *p* = 0.0431
PE	11.84 ± 1.18	7.03 ± 0.71 ^a^	a; *p* < 0.0001	8.54 ± 0.80^x^	x; *p* = 0.0433
PC	14.14 ± 1.44	6.80 ± 1.04 ^a^	a; *p* < 0.0001	7.39 ± 1.08 ^a^	a; *p* = 0.0167

Data points represent the mean ± SD; a—statistically significant differences vs. healthy subjects; x—statistically significant differences vs. patients with psoriasis vulgaris.

**Table 3:** The value of total concentrations of negatively (C_TA_) and positively (C_TB_) charged groups and their association constants with H^+^ (K_AH_), and OH^-^ (K_BOH_) ions of blood cells (erythrocytes, lymphocytes and granulocytes) as well as skin cells (keratinocytes and fibroblasts) of patients with psoriasis vulgaris (*n* = 16) and psoriatic arthritis (*n* = 8) as well as healthy subjects (*n* = 8).

Groups	Parameters							
	C_TA_ (10^−6^ mol/m^2^)		C_TB_ (10^−6^ mol/m^2^)		K_AH_ (10^2^ m^3^/mol)		K_BOH_ (10^7^ m^3^/mol)	
**Erythrocytes**								
Control	5.87 ± 0.22		1.45 ± 0.11		0.74 ± 0.08		7.86 ± 0.24	
Psoriasis vulgaris	6.09 ± 0.41		1.92 ± 0.16 ^a^	a; *p* = 0.0036	0.67 ± 0.07		8.11 ± 0.32	
Psoriatic arthritis	6.47 ± 0.42 ^a^	a; *p* = 0.0173	2.09 ± 0.17 ^a^	a; *p* < 0.0001	0.59 ± 0.06 ^a, x^	a; *p* = 0.0016 x; *p* = 0.0486	8.41 ± 0.31 ^a^	a; *p* = 0.004
**Lymphocytes**								
Control	1.56 ± 0.11		0.99 ± 0.08		0.42 ± 0.04		2.35 ± 0.10	
Psoriasis vulgaris	3.65 ± 0.37 ^a^	a; *p* = 0.0085	1.92 ± 0.19 ^a^	a; *p* < 0.0001	0.62 ± 0.06 ^a^	a; *p* = 0.0004	2.71 ± 0.18 ^a^	a; *p* = 0.0001
Psoriatic arthritis	4.77 ± 0.46 ^a, x^	a; *p* < 0.0001 x; *p* = 0.0126	1.78 ± 0.18 ^a^	a; *p* = 0.0229	0.64 ± 0.07 ^a^	a; *p* = 0.0007	2.66 ± 0.15 ^a^	a; *p* = 0.0043
**Granulocytes**								
Control	1.06 ± 0.10		1.45 ± 0.12		0.43 ± 0.05		6.16 ± 0.55	
Psoriasis vulgaris	4.04 ± 0.41 ^a^	a; *p* = 0.0007	2.62 ± 0.25 ^a^	a; *p* < 0.0001	0.64 ± 0.08 ^a^	a; *p* = 0.0051	8.67± 0.89 ^a^	a; *p* = 0.0007
Psoriatic arthritis	4.40 ± 0.45 ^a^	a; *p* = 0.0003	1.89 ± 0.18 ^x^	x; *p* = 0.0117	0.79 ± 0.08 ^a, x^	a; *p* < 0.0001 x; *p* = 0.0489	8.93 ± 0.92 ^a^	a; *p* = 0.0004
**Keratinocytes**								
Control	2.96 ± 0.24		0.79 ± 0.08		0.53 ± 0.06		6.16 ± 0.38	
Psoriasis vulgaris	4.93 ± 0.48 ^a^	a; *p* < 0.0001	1.86 ± 0.19 ^a^	a; *p* < 0.0001	0.68 ± 0.07 ^a^	a; *p* < 0.0001	7.14 ± 0.59 ^a^	a; *p* = 0.0001
**Fibroblasts**								
Control	3.33 ± 0.30		0.50 ± 0.04		0.48 ± 0.04		7.05 ± 0.41	
Psoriasis vulgaris	5.04 ± 0.52 ^a^	a; *p* < 0.0001	0.64 ± 0.07 ^a^	a; *p* < 0.0001	0.18 ± 0.04 ^a^	a; *p* < 0.0001	8.09 ± 0.62 ^a^	a; *p* = 0.0033

Data points represent the mean ± SD; a—statistically significant differences vs. healthy subjects; x—statistically significant differences vs. patients with psoriasis vulgaris.

## Supplementary Materials

The following are available online at https://www.mdpi.com/1422-0067/21/23/9129/s1.

## Abbreviations

4-HNE	4-hydroxy-2-onenal
ANOVA	Analysis of variance
CB	Cannabinoid receptor
CBD	Cannabidiol
C_TA_	Total concentrations of negatively
C_TB_	Total concentrations of positively
EDTA	Ethylenediaminetetraacetic acid
GC	Gas chromatography
GD	Disialoganglioside
GT	Trisialoganglioside
HPLC	High performance liquid chromatography
IL	Interleukin
JAK-STAT	Janus Kinase and Signal Transducer and Activator of Transcription
K_AH_	Association constants with hydrogen ions
K_BOH_	Association constants with hydroxide ions
LPA	Lysophosphatidic acid
LPC	Lysophosphatidylcholine
MAPK/AP	MAP kinase activated protein kinase
MDA	Malondialdehyde
Nrf2	Nuclear factor (erythroid-derived 2)-like-2 factor
NFkB	Nuclear factor kappa-light-chain-enhancer of activated B cells
PA	Phosphatidic acid
PBS	Phosphate-buffered saline
PC	Phosphatidylcholine
PE	Phosphatidylethanolamine
PI	Phosphatidylinositol
PLA2	Phospholipase A2
PS	Phosphatidylserine
PUFAs	Polyunsaturated fatty acids
ROS	Reactive oxygen species
SM	Sphingomyelin
TH	T helper lymphocytes
